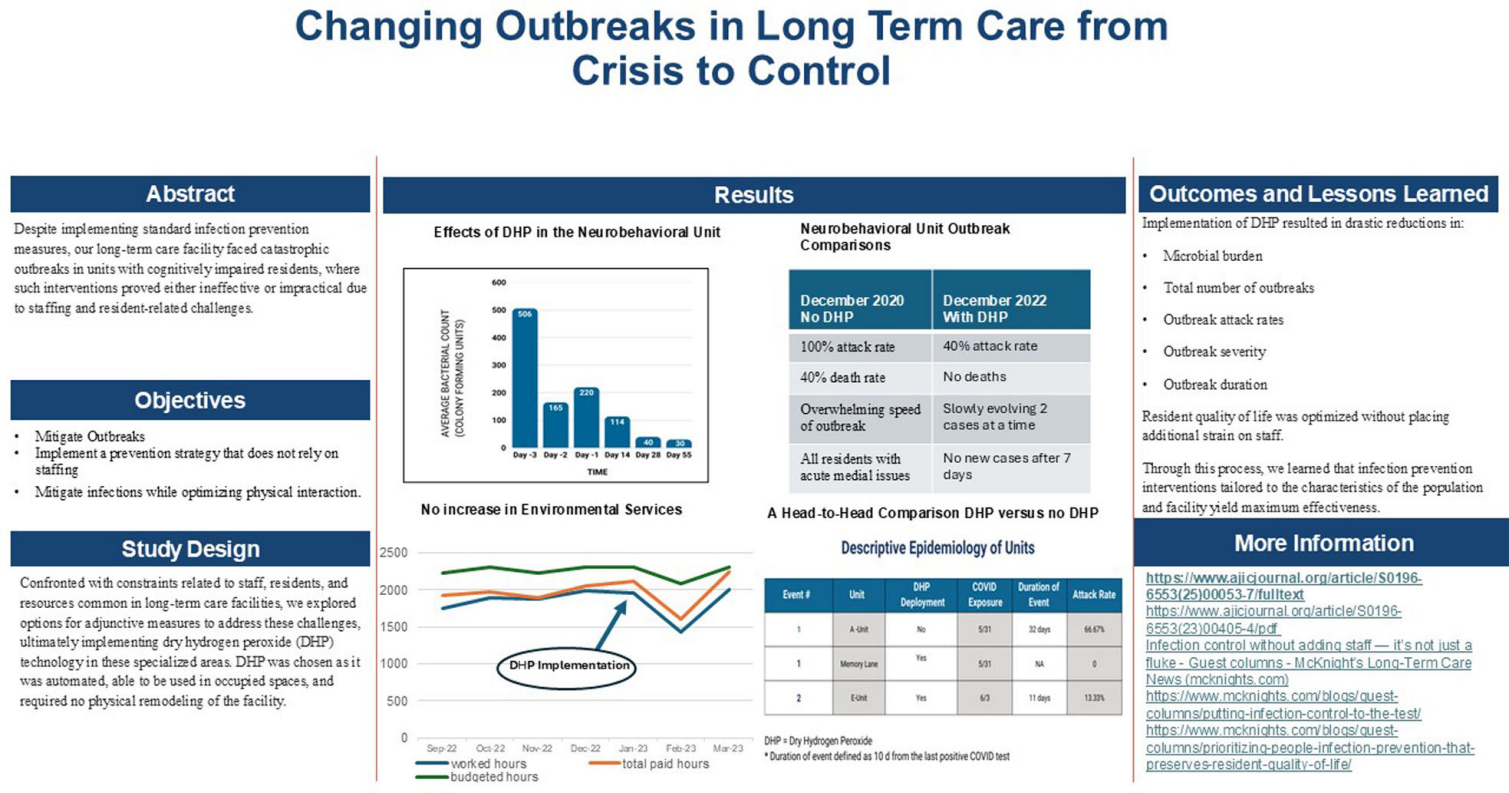# 289 Implementation Gaps in Staphylococcus aureus Screening and Mupirocin Decolonization Among Hospitalized Patients With Central Lines

**DOI:** 10.1017/ash.2026.10648

**Published:** 2026-06-23

**Authors:** Mary Cole

**Affiliations:** 1 The Highlands at Brighton

## Abstract

**Background:** Despite implementing standard infection prevention measures, our long-term care facility faced catastrophic outbreaks in units with cognitively impaired residents, where such interventions proved either ineffective or impractical due to staffing and resident-related challenges. Project Design: Confronted with constraints related to staff, residents, and resources common in long-term care facilities, we explored options for adjunctive measures to address these challenges, ultimately implementing dry hydrogen peroxide technology in these specialized areas. Outcomes and Lessons Learned: This intervention resulted in drastic reductions in microbial burden, the total number of outbreaks, outbreak attack rates, severity, and duration, while optimizing resident quality of life without placing additional strain on staff. Through this process, we learned that infection prevention interventions tailored to the characteristics of the population and facility yield maximum effectiveness.

**Figure:**